# PyTMs: a useful PyMOL plugin for modeling common post-translational modifications

**DOI:** 10.1186/s12859-014-0370-6

**Published:** 2014-11-28

**Authors:** Andreas Warnecke, Tatyana Sandalova, Adnane Achour, Robert A Harris

**Affiliations:** Department of Clinical Neuroscience, Karolinska Institutet, Center for Molecular Medicine, Applied Immunology & Immunotherapy, L8:04, Karolinska Hospital, SE-171 76 Stockholm, Sweden; Department of Medicine Solna, Science for Life Laboratory, Karolinska Institutet, Stockholm, Sweden

**Keywords:** Post-translational modifications, PyMOL plugin, Structural bioinformatics, Modeling, Acetylation, Carbamylation, Citrullination, Oxidations, Malondialdehyde adducts, Nitration

## Abstract

**Background:**

Post-translational modifications (PTMs) constitute a major aspect of protein biology, particularly signaling events. Conversely, several different pathophysiological PTMs are hallmarks of oxidative imbalance or inflammatory states and are strongly associated with pathogenesis of autoimmune diseases or cancers. Accordingly, it is of interest to assess both the biological and structural effects of modification. For the latter, computer-based modeling offers an attractive option. We thus identified the need for easily applicable modeling options for PTMs.

**Results:**

We developed PyTMs, a plugin implemented with the commonly used visualization software PyMOL. PyTMs enables users to introduce a set of common PTMs into protein/peptide models and can be used to address research questions related to PTMs. Ten types of modification are currently supported, including acetylation, carbamylation, citrullination, cysteine oxidation, malondialdehyde adducts, methionine oxidation, methylation, nitration, proline hydroxylation and phosphorylation. Furthermore, advanced settings integrate the pre-selection of surface-exposed atoms, define stereochemical alternatives and allow for basic structure optimization of the newly modified residues.

**Conclusion:**

PyTMs is a useful, user-friendly modelling plugin for PyMOL. Advantages of PyTMs include standardized generation of PTMs, rapid time-to-result and facilitated user control. Although modeling cannot substitute for conventional structure determination it constitutes a convenient tool that allows uncomplicated exploration of potential implications prior to experimental investments and basic explanation of experimental data. PyTMs is freely available as part of the PyMOL script repository project on GitHub and will further evolve.

**Graphical Abstract:**
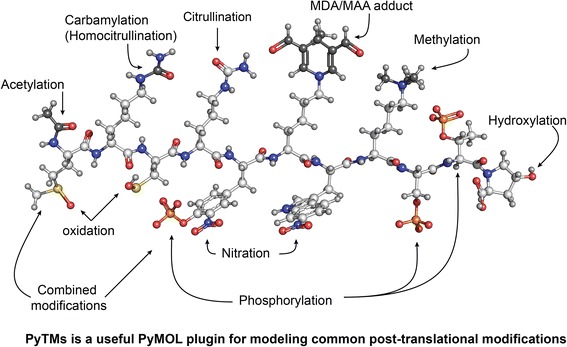
PyTMs is a useful PyMOL plugin for modeling common post-translational modifications.

**Electronic supplementary material:**

The online version of this article (doi:10.1186/s12859-014-0370-6) contains supplementary material, which is available to authorized users.

## Background

### Post-translational modifications

Post-translational modifications (PTMs) are chemical alterations that occur after protein synthesis [1,2]. Physiologically, PTMs are selectively introduced by specific, dynamically-regulated enzymes in order to alter the target protein’s biochemical properties. PTMs are thus employed to regulate biological processes by altering, for example, protein activity, signaling, localization, conformation, binding or turnover [3-5]. The involvement of PTMs in cellular signaling cascades, particularly kinase-mediated phosphorylation, is being extensively researched.

Conversely, pathophysiological PTMs are associated with episodes of oxidative imbalance, and there is a constant cellular endeavor to prevent and repair their occurrence. Unlike physiological PTMs, pathophysiological PTMs typically occur in proximity to the origin of the modifying agent in an uncontrolled fashion. The formation of such pathophysiological PTM on their target proteins may impinge on cellular homeostasis and immune recognition [3-8]. Smoking in particular has been identified as a risk factor with respect to PTMs and inflammation [9-11]. Due to altered immunogenicity and the associated inflammation, pathophysiological PTMs are of particular interest in the context of autoimmune pathogenesis [3-5]. Conversely, the PTM-mediated altered immunogenicity of proteins has been suggested as a means of escape from immune surveillance in cancer [7].

As implicated above, the effect that a PTM exerts on its carrier protein is highly individual and context-dependent [3,4,12,13]. Regarding a protein’s activity, modification can be regulatory, resulting in either gain- or loss-of-function, or alternatively have no immediate effect - depending largely on the location of the PTM. The latter is argued to have been evolutionarily selected for in the context of Methionine oxidation, in particular in mitochondrial enzymes, because oxidation of the distal ‘scavenger’ residues protects critical functional sites [14]. Taken together, this highlights the relevance of structural positioning in regard to PTMs.

There are accumulating examples of specific PTMs being recognized by scavenger receptors, complement components and antibodies due to alterations in structure and charge [12,15-17]. One sensible biological function is the accelerated disposal of damaged proteins. Conversely, a classic example is the accumulation of ‘foam cells’ in atherosclerotic plaques. These are macrophages that have accumulated oxidized low density lipoprotein due to increased uptake mediated by the scavenger receptor CD36 [16]. More recently, complement factor H (CFH) has been demonstrated to be involved in the disposal of oxidatively modified proteins bearing adducts jointly formed by Malondialdehyde (MDA) and Acetaldehyde (MAA-adduct) [15]. MDA is a highly reactive three-carbon dialdehyde formed during lipid peroxidation. A polymorphism in CFH (HIS instead of TYR at residue 402) is argued to result in a reduced clearance of damaged MAA-modified proteins in the eye, with causative links to age-related macular degeneration [15]. Interestingly, native antibodies from newborns appear to recognize the same modification [17]. The structural nature of this and other such interactions, however, remain to be characterized in detail.

The antigenicity of complement-bound antigen has been demonstrated to increase by several orders of magnitude, as evidenced by co-ligation of the B cell receptor and CD19 bridged by complement component C3dg [18]. This agrees with the reported antigenicity of MDA-adducted proteins [19,20], provided that pro-inflammatory complement components recognize PTMs and thus ‘flag’ them for increased immune surveillance. Furthermore, MDA has been experimentally implied in the pathogenesis of animal models of Multiple Sclerosis (MS) [5,21], and there is clinical evidence of elevated lipid peroxidation as a source of MDA in MS patients [22,23].

In fact, most autoimmune diseases have an implied involvement of PTMs, and associated altered peptide ligands (APLs) have been identified [3-5,24,25]. A common mechanistic denominator in autoimmunity (or allergy) is the absence of negative selection of T or B cells during education. Such non-tolerized, cross-reactive clones, capable of recognizing ‘modified self’ or ‘cryptic epitopes’, are suspected to initiate the break of immunological tolerance [3,4]. The key underlying molecular events are the processing of the non-tolerized epitope for presentation, and finally the recognition of this peptide presented within a matching Major Histocompatibility Complex (MHC) by a cross- and/or self-reactive T cell receptor (TCR).

In Rheumatoid Arthritis there is a strong association of specific MHC alleles and their ability to present citrullinated APLs, i.e. epitopes that have undergone enzymatic deimination of arginine residues to citrulline. The molecular basis of this association has only recently been characterized at a structural level [25]. There are accumulating examples of APLs being either superior or inferior epitopes in the context of MHC presentation or TCR recognition [12,24,25]. Taken together, these emphasize the requirement for individual structural assessment in the context of immunology and PTMs.

In conclusion, PTMs constitute a complex layer in protein biology and immune recognition. The diversity and context-dependence of PTM-mediated effects calls for individual assessment at a structural level. Furthermore, we identify an unmet need for easily applicable modeling tools in respect to PTMs.

### Post-translational modifications in protein models

**Figure 1:**
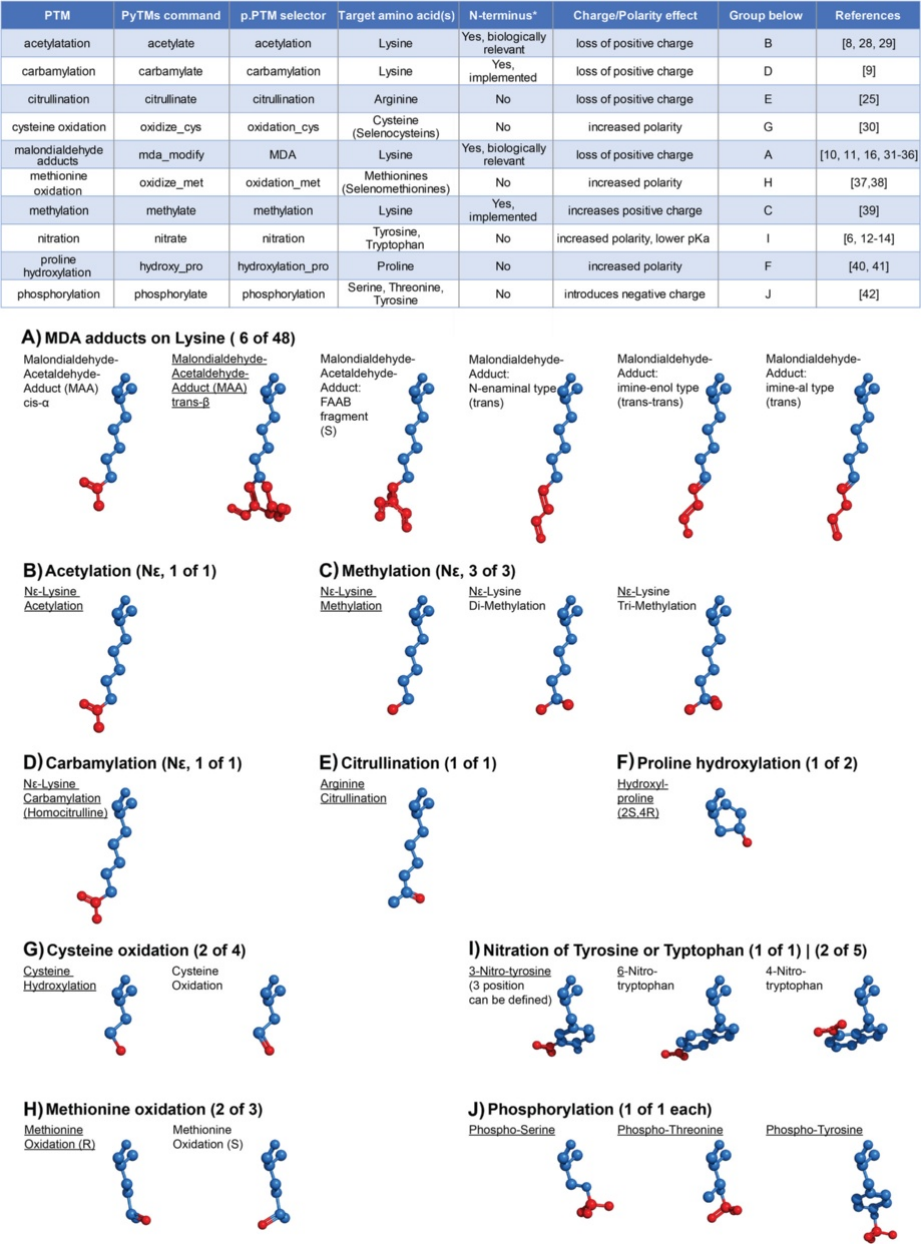
**Overview.** This figure gives an overview over all currently covered PTMs that can be introduced in PyMOL using PyTMs. Top: A table of the possible modifications in alphabetical order. PTM = post-translational modification. The columns from left to right contain: name of modification, the PyTMs command that is used to introduce it in the PyMOL API (when not using the GUI menu), a custom property selector name through which the modification can be selected in PyMOL (requires incentive PyMOL version 1.7 or higher), the target amino acid, information on whether N-termini can be modified using the respective function (*= modifiable, excluding Proline), a summary of charge effects, a group indicator (see below) and lastly selected references. Bottom: A depiction of representative modifications from all classes, grouped by amino acid and modification type. Note that the individual panels** A**-**J** correspond to the ‘group below’ column of the above table. All residues are aligned by the amino acid head, the terminal carboxyl group facing towards the viewer. The base is colored blue and the PTMs in red. Hydrogens are omitted. Groups are labeled as in the table above. For each category the displayed and total number of variants are indicated. The respective defaults for categories are underlined. Configurational diastereomers are indicated with the R or S nomenclature.

As outlined above, it is of increasing interest for researchers to assess the potential impact of PTMs of particular proteins, or to provide structural insight into how an introduced PTM potentially can exert an experimentally observed effect. Recent advances in accounting for PTMs during resolution of 3D structures have been made [26]. However, resolving a 3D-structure may prove unattractive or infeasible due to financial, technical or time-related reasons. In this case, modeling the PTM *in silico* provides a rapid alternative. The online resource PhosphoSitePlus (http://www.phosphosite.org/) [27] is able to indicate relevant sites in proteins by mining database entries, but does not include or introduce the PTM unless it is already present in an existing crystal structure. To our knowledge, no simple, freely available tool is available that can easily perform such a task. Manual structure editing is conceivable using the visualization software PyMOL (http://www.pymol.org/), but is likely time-consuming and will thus not provide good flexibility for various models. We therefore developed PyTMs as an applicable PyMOL plugin that enables the straightforward introduction and modeling of a set of PTMs into existing 3D-structures. PyMOL is commonly used, easy to handle and freely available. Advantages of using automated PTM creation include: firstly, standardized and reproducible PTMs; secondly, minimal time-requirement; thirdly, availability of the option to a broader user audience. PyTMs currently encompasses ten common PTM categories which are summarized in Figure 1, namely acetylation [8,28,29], carbamylation [9], citrullination [25], cysteine oxidation [30], malondialdehyde adducts [10,11,16,31-36], methionine oxidation [37,38], methylation [39], nitration [6,12-14], proline hydroxylation [40,41] and phosphorylation [42].

## Implementation

### PyTMs implementation notes

We implemented PyTMs as a plugin for the PyMOL Molecular Graphics System (Schrödinger, LLC., http://www.pymol.org/). The name PyTMs has been chosen as an analogy to PyMOL, Python (https://www.python.org/) and PTMs. PyTMs is written in Python and is open source: the file is freely available as part of the PyMOL script repository, allowing version control and updating (visit http://www.pymolwiki.org/index.php/Pytms for more information). The first release version is provided as Additional file 1. PyTMs is designed to facilitate the introduction of post-translational modifications into existing 3D-structure models. The intention is to provide a simple research tool that allows making predictions and explaining observations associated with PTMs, yet requiring only basic PyMOL user experience. For this reason we also provided a convenient user interface (cf. Additional file 2).

### PyTMs conception

PyTMs allows modeling of a selected set of common modifications. We used RCSB database (http://www.rcsb.org/) entries containing modified residues as templates where available. Furthermore, the nomenclature of modified residues was adjusted to correspond to this database as far as possible. More detailed information on the implementation of certain PTMs is available in Additional file 3. Though PyTMs employs basic structure editing options already available in PyMOL, the advantage of using PyTMs is the automation of the process, which enables basic users to edit any structure within seconds and currently does not depend on a library.

### Basic algorithm

The basic algorithm of PyTMs follows a pattern that is similar for each individual PTM. First, the input selection is broken down to filter candidate amino acids as determined by the class of PTM and settings. If selected, this will involve a pre-selection of surface-exposed residues, based on a provided solvent-accessible area cutoff (in Å^2^). The subsequent sub-selection of target atoms for modification is automatic, thus the user does not need to provide a specific selection, apart from the object or atom group. Selections or objects can also be chosen from the integrated menu. The candidate residues are filtered for existing modifications and sequentially modified, renamed, adjusted and optionally colored.

For some PTMs, e.g. Malondialdehyde adducts, we implemented a residue-based basic structure optimization. This optimization avoids steric Van-der-Waals (vdW) clashes by rotating the residue into a favorable position. The calculated vdW strain is minimized in the process. For reduction of calculation time the strain calculation is performed locally per residue. There is an option to probe baseline strain increase and thus avoid calculation loops for unfavorable positioning of the original residue. The optimization and/or steric clashes can be visualized in PyMOL (cf. Additional file 4).

### Further information

Practical information, such as a user guide and application notes, are available at the PyTMs wiki page (http://www.pymolwiki.org/index.php/Pytms) or by accessing each function’s specific help, e.g. from the plugin menu. Users have the option of using either the menu and/or the Python function that extend the PyMOL API, e.g. for use in scripts. PyTMs has been developed and tested using the current PyMOL incentive version 1.7, but is compatible with version 1.3. Older versions and open-source PyMOL have minor restrictions, the main one being the lacking support of custom property ‘p.PTM’ selectors. The overall functionality is, however, otherwise not affected. Modified models can be saved as ‘.pkl’ files, which preserve bonds and valences correctly. We anticipate that additional PTMs and functionality will be added as PyTMs becomes updated in due time.

### Application examples

Here we provide some basic application examples of how PyTMs can be employed to address research questions related to PTMs, which we discuss below in the Results section:

### Enzyme inhibition: nitrated HPR1

**Figure 2:**
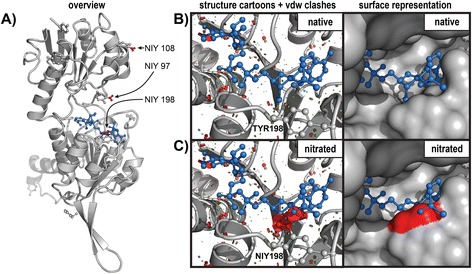
**Example – inhibition of HPR1 by nitration.** This figure is an application example of using PyTMs in the setting of enzyme inhibition. Briefly, the model of hydroxypyruvate reductase 1 from *Arabidopsis thaliana* was nitrated at three specific Tyrosine residues using PyTMS as described in the implementation section. This enzyme has previously been demonstrated to be nitrated at these residues and thereby functionally inhibited. NIY = Nitrotyrosine. **A)** An overview of the nitrated enzyme monomer is depicted in secondary structure cartoon representation. Additionally, the locations of (Nitro-)Tyrosine residues have been highlighted in stick representation. The nitro groups in residues 97, 108 and 198 are colored red. The co-factor (NADPH) in stick representation is colored blue. **B + C**) A close-up view of the co-factor (NADPH) binding site in the enzyme. **B**: native; **C**: nitrated. Left: Representation as cartoon with the (nitrated) TYR198. Steric clashes based on vdW overlap are represented by the colored discs. The increasing size and redness of the discs correlate with stronger vdW strain. The native enzyme does not have significant steric clashes. The nitration, however, introduces significant steric clashing with the co-factor and occludes the binding site. Right: identical view as in the left image, but depicted as surface with the extent of the nitration colored red. In conclusion, the denied binding of co-factor, mainly due to nitration at TYR198, is the structural basis for impaired enzyme activity.

The model of hydroxypyruvate reductase 1 (HPR1) from *Arabidopsis thaliana* was kindly provided by Dr. Francisco J. Corpas [43]. The missing nicotinamide adenine dinucleotide phosphate (NADPH) co-factor was added from the aligned structural homologue *Pyrococcus horikoshii* hydroxypyruvate reductase [PDB: 2DBQ] and slightly adjusted to fit the cavity without significant steric clashes. Using PyTMs, we introduced nitrations of Tyrosine residues that have previously been experimentally determined to be nitrated [43]. In particular, nitration of TYR198 close to the active site has been demonstrated to inhibit enzyme activity. The steric vdW clashes in HPR and the NADPH co-factor (Figure 2B + C) were visualized using an adapted version of the PyMOL script ‘show_bumps’ (available on the PyMOL wiki), which has been integrated within PyTMs. This visualization of vdW clashes can be performed directly in conjunction with modification. Alternatively, clashes can be visualized in unmodified protein or retrospectively by using a dedicated function.

### SWISS modeling and multiple modifications: oxidation of hERG1

**Figure 3:**
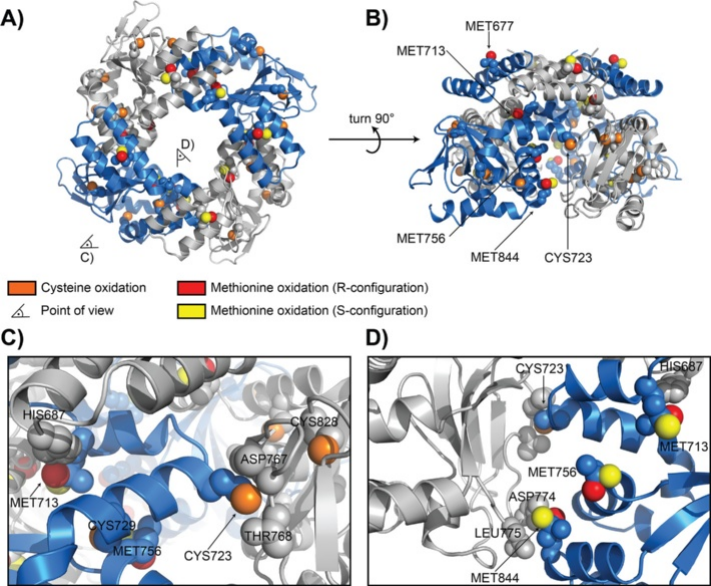
**Example – oxidation of the hERG potassium channel.** This figure is an application example of using PyTMs in conjunction with SWISS modeling, introducing multiple modifications and demonstrating the possibility of modeling stereochemical variants. As the structure of hERG is only partially resolved, a complete model of the intracellular C-terminal domain of hERG1 (residues 667–865) was generated as described in the implementation using SWISS modeling. Using PyTMs, this model was then doubly modified by cysteine oxidation and methionine oxidations. Selected residues are displayed as spheres. Methionine oxidation was performed on object copies introducing either the configurational Methionine sulfoxide R-isomer (red oxygen) or the S-isomer (yellow oxygen). Note that these configurational isomers are superimposed to highlight the differential positioning and do not coexist on the same residue. Cysteine oxidation is not racemic (orange oxygen). Oxidation of hERG1, especially at CYS723, has been previously demonstrated to result in an accelerated deactivation of the associated ion channel. **A)** An overview of a homo-tetramer of hERG1 intracellular C-terminal domains, as viewed from above i.e. from the cell membrane or pore. The equivalent monomers are colored either blue or light gray for better distinction. The viewing positions for C) and D) are indicated. **B)** This view corresponds to A, but the complex is viewed from the side. Selected residues have been labeled, as indicated. Note the critical cysteine 723 at the dimer interface. **C)** A close-up view of the critical oxidized CYS723 at the dimer interface viewed from outside the complex (cf. A). Note how the polar oxygen extends the range of the cysteine residue to interfere with the adjacent loop (ASP767 and THR768). Expectedly, a resulting increase in steric displacement can explain the previously reported increase in deactivation rate. **D)** A close-up view from inside the complex focusing on adjacent Methionine sulfoxide residues.

As the structure of human *ether à go-go-*related gene 1 (hERG1, K_V_11.1, KCNH2) is only partially available, we applied SWISS homology modeling [44] using HCN2-I [PDB: 3BPZ] [45] as template to generate a joined model of the hERG1 intracellular C-terminal region (residues 667–865), including the C-linker and regulatory domains. Using PyTMs we oxidized Cysteines and Methionines, either in R- or S- configuration, and superimposed the results for comparison (Figure 3).

### MAA-modification of Bovine Serum Albumin surface-exposed lysines

Here we exemplify the integration of PyTMs with sub-selections and the possibility to predict surface-restricted modifications, and basic optimization. Pre-selection of surface-exposed atoms is managed by a cutoff using the calculated solvent-accessible surface area for each atom. This pre-selection is set by a user-provided cutoff and is integrated in PyTMs. The original procedure is described on the PyMOL wiki (‘findSurfaceResidues’). Here, we restricted modification to surface-exposed Lysine epsilon amine groups in a model of Bovine Serum Albumin (BSA, [PDB: 4F5S]), using a cutoff of 25 Å^2^. This cutoff yielded a reasonable selection of exposed atoms that were modified using PyTMs. We introduced the default Malondialdehyde-acetaldehyde (MAA) adduct (cf. Additional file 3). An optimization level of 3 was applied to account for potential steric overlap of the bulky adducts.

### Altered peptide ligands – MHC complexes

Crystal structures of the immunodominant lymphocytic choriomeningitis virus (LCMV)–derived epitope gp34 in complex with a murine Major Histocompatibility Complex (MHC, H-2K^b^) have previously been determined for the native epitope [PDB: 3ROO] and a nitrated escape variant [PDB: 3ROL] [24]. Starting with the native unmodified variant we nitrated the peptide ligand *in silico* and sterically refined the structure. First we tested alternative ortho-positions for the nitration (rotamer of 3-Nitrotyrosine). We thereafter chose alternative backbone-dependent rotamers for GLU152 in the MHC side chain and TYR116 in the beta-sheet of the MHC heavy chain peptide-binding cleft. These two adaptations were expected to accommodate the nitro group without significant steric clashes. Finally, we aligned the resulting models with the experimentally resolved structure containing the nitrated epitope. The alignment demonstrated that the model created using PyTMs corresponded well to the previously determined crystal structure [24].

## Results and discussion

### Straightforward modeling of post-translational modifications

The purpose of PyTMs is to facilitate modeling of PTMs. There is a steady interest from various fields in the impact of PTMs and hence a need for tools that enable their prediction and analysis. Although basic structure editing options are available in the PyMOL base package, we identified two hurdles. Firstly, manual editing requires additional user experience and secondly, it can be laborious even when using libraries. We therefore set out to facilitate the procedure by developing a plugin.

We implemented ten different PTMs as listed in Figure 1. These cover a large ensemble of common PTMs, all of which are well-studied and implicated either in important physiological processes or in disease settings (cf. Figure 1). Here we provide a selection of examples and discuss the overall applicability of PyTMs as a plugin tool.

### Application example: enzyme inhibition in nitrated HPR1

We searched the literature for examples of PTMs affecting enzymatic activity. For instance, Corpasa *et al.* demonstrated that nitration of TYR198 in hydroxypyruvate reductase 1 from *Arabidopsis thaliana* results in enzyme inhibition [43]. Using site-directed mutagenesis the authors confirmed that nitration of TYR198 in the active site is responsible for the loss of function, but they did not include nitrations in the structural model. Using the PyTMs plugin we introduced nitration at the relevant sites and investigated the model in further detail. Consistent with the previously reported observations, we demonstrate that nitration of TYR198 obstructs the co-factor binding site, providing a structural explanation for reduced enzyme activity (Figure 2).

This example demonstrates that PyTMs can be utilized to help explain experimental observations on the basis of a structural model and suggest that it can also be used to predict such loss of function.

### Application example: multiple oxidation of hERG1

The human *ether à go-go-*related gene 1 hERG1 (K_V_11.1, KCNH2) encodes a potassium channel infamous for off-target drug side-effects that result in cardiac arrhythmias. Structurally hERG1 resembles other members of hyperpolarization-activated, cyclic nucleotide-modulated (HCN) channels [45]. The pore-forming transmembrane domains are connected to an intracellular C-linker region and a regulatory ligand-binding domain. Both the C-linker and the ligand-binding domain contain residues that are involved in homo-tetramerization of the intracellular domains. This structured tetramerization is regarded as the underlying mechanism towards opening the associated potassium channel upon ligation [45]. The intracellular region, in particular the C-linker, is sensitive to oxidation of Methionine [46] and/or Cysteine [30] residues. These oxidations account for an accelerated channel deactivation [30,46].

Our extended model in Figure 3 supports the previously reported conclusions. The generated model supports a critical role for oxidation of CYS723 at the dimer interface, which is consistent with a faster deactivation of hERG1 following oxidation due to increased structural strain. The same argument holds for Methionine 844. The oxidized MET844 has proximity to the dimer interface and sterically clashes with ASP774 and LEU775 of the neighboring chain. As MET713 or MET756 do not have immediate proximity to neighboring subunits, the inhibitory effect may involve rearrangement of the associated helices upon oxidation of these residues, and therefore a more indirect mode of inhibition. Note that the alternative configurational isomers (R:red, S: yellow) may have unequal impact in this respect.

Based on this model the critical role of CYS723 in the susceptibility of hERG1 to oxidation derives from its open accessibility and positioning at the dimer interface. The equivalent explanation is valid for MET844. For the remaining Methionine sulfoxides the inhibition is expected to be less direct and may involve rearrangement of the associated C-linker helices. Hence, several Methionine residues may require oxidation to confer the same degree of inhibition as oxidation of CYS723 or MET 844 alone.

This example alludes to several modeling aspects. First, it exemplifies the use of homology modeling [44] in case a resolved structure is unavailable. Secondly, the model is an example of introducing several different PTMs into one protein model. In fact, the only condition for consecutive modification *in silico* using PyTMs is that the target atom is still available. For instance, it is possible to co-modify an N-terminal Tyrosine by phosphorylation, nitration and N-terminal acetylation (see also graphical abstract, Additional file 5). This may not necessarily be biologically relevant, as nitration has been demonstrated to impede subsequent enzymatic phosphorylation [47], but this is allowed within the program. Finally, this example addresses modeling of diastereomers [48]. We implemented biologically relevant diastereomers for Methionine sulfoxide, Cysteine-s-dioxides and the 2-formyl-3-(alkylamino)-butanal (FAAB fragment) adduct of Malondialdehyde. Hydroxyl-proline is technically chiral but biologically always present in a 4-R configuration, since this is an enzymatic modification. However, the option for a 4-S variant exists in PyTMs. As diastereomers of Methionine sulfoxide are repaired by distinct enzymes [38] we suspect that additional biomolecules such as antibodies, T-cell receptors, complement components and scavenger receptors also discriminate stereospecific variants [48].

Notably, this example also indicates a current limitation of the plugin, namely a disregard for potential global conformational changes induced by PTMs. Although some manual local refinement can be informative, larger rearrangements will become increasingly artificial and unreliable. Conformational changes that affect large or distal areas will be especially problematic. With this in mind conventional structure determination, although laborious, will yield more conclusive results. Here we point out SWISS modeling [44] as a previously available resource that can be applicable in case of unavailable crystal structures.

In summary, it is possible to induce several modifications in one model and specify stereospecific variants. The resulting model should, however, not be over-interpreted as potential global conformational changes are not handled by the software. Nonetheless, it is possible to explain experimental data or make predictions at a detailed structural level using PyTMs when respecting these caveats.

### Application example: MDA-modification of Bovine Serum Albumin surface-exposed Lysines

A critical factor that influences the likelihood of modification is accessibility of the target atom. Non-selective oxidative modifications, such as Malondialdehyde (MDA) derived from lipid peroxidation, will primarily target the most accessible residues while buried residues are likely spared. Using PyTMs we selected only surface-exposed epsilon-amines (area cutoff: 25 Å^2^) in a model of BSA and introduced Malondialdehyde-acetaldehyde (MAA) adducts. We implemented a basic vdW strain optimization for this PTM category as many adducts are comparatively bulky and tend to sterically clash with adjacent residues. Applying optimization rotates the modified Lysine to a more favorable rotamer, but requires some calculation time. We implemented that this optimization can be animated in PyMOL (cf. Additional file 4). Additional information on the implementation of MDA adducts is provided in the Additional file 2 [48,49].

**Figure 4:**
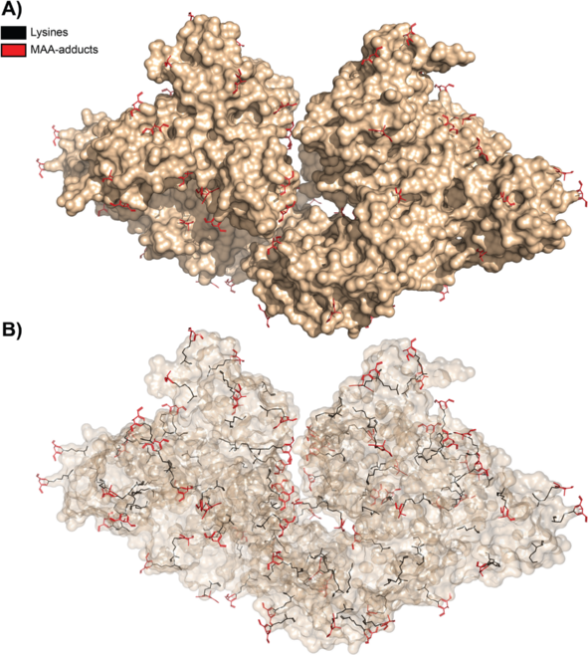
**MAA adducts on the surface of BSA.** This figure is an application example of using PyTMs to selectively modify only solvent-accessible residues. As described, a model of Bovine Serum Albumin (BSA) was used to sub-select surface atoms before introducing Malondialdehyde-acetaldehyde (MAA) adducts. The brown surface corresponds to the original unmodified BSA. Both unmodified Lysines (43/118) and MAA-Lysines (75/118) are depicted in stick representation, but colored black or red, respectively. **A)** An overview of MAA-modified BSA with opaque surface. Note the surface decoration by MAA adducts (red sticks). **B)** An identical image with transparent surface. Note how the inaccessible/unmodified Lysines (black sticks without adduct) are likely to be shielded from modification within the protein’s interior volume. Additional MAA-modified-Lysines from the protein’s distal surface are also visible.

Figure 4 displays MAA-modified BSA in which the surface-accessible amines have been modified (75 of 118 Lysines). The pre-selection noticeably restricted modification to surface residues while inaccessible Lysines remained unmodified (Figure 4B). Indeed, buried residues are most likely protected from modification within the protein, unless it unfolds in the process. Unlike the first example, in which the nitrations were detected by Mass-Spectrometry prior to modeling, this example is predictive. Notably, should Mass-Spectrometry detect modified residues at unexpected locations, this may indicate that the protein becomes unfolded during the modification process. In relation to topology we were unable to assess the associated charge impact of modification. The limitation is currently that force field applications such as the Adaptive Poisson-Boltzmann Solver (APBS) [50] currently only support standard amino acids. This aspect may be addressed in the future, but will require the generation of custom force fields for PTM amino acids. Experimental data indicates that increasing MAA modification gradually eliminates positive charge and thus alters the migration of proteins in native or isoelectric focusing gels ([36] and data not included).

In summary, we conclude that restricting modification to experimentally defined or accessible atoms are feasible approaches to improve model accuracy. Optimally, each model should be generated according to supporting experimental data at hand or be experimentally validated accordingly. We point out that PyTMs could for instance be used to introduce missing PTMs for structure information retrieved from PhosphoSitePlus [27].

### Application example: MHC-restricted peptide epitopes

Modifications can be introduced into single amino acids, peptides or whole proteins using PyTMs. In the context of altered peptide ligands (APLs) and Major Histocompatibility Complexes (MHCs), modeling can be useful to explain or predict whether an APL can be presentable by a respective MHC allele and/or how the introduced PTM and APL may be oriented.

Herein we made use of a pair of crystal structures of murine H-2K^b^ MHCs in complex with the lymphocytic choriomeningitis virus (LCMV)-derived immunodominant epitope gp34, without or with nitration [24]. The nitrated APL is an escape variant and exhibits significantly reduced binding affinity [24]. Using the native complex we nitrated the Tyrosine at position 3 in gp34 and performed minor local refinements to accommodate the APL.

**Figure 5:**
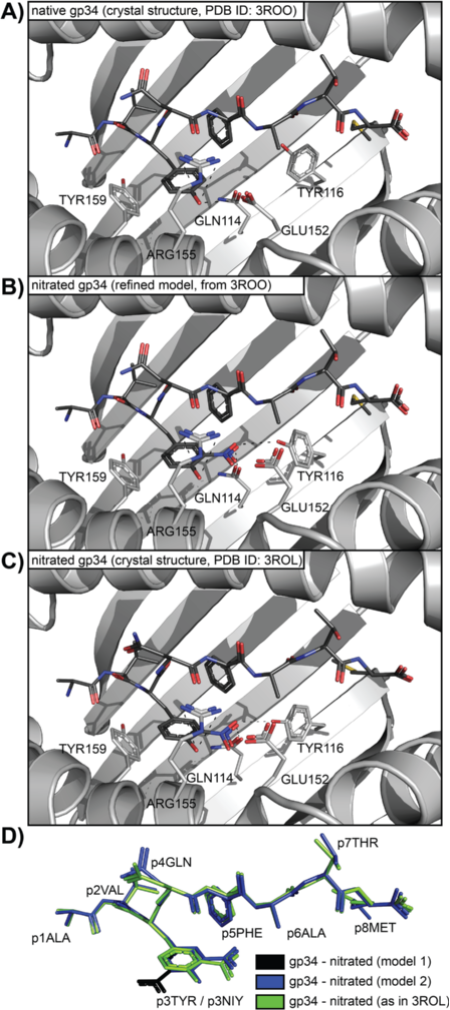
**MHC–related applications.** In this figure we tested the applicability of PyTMs with respect to altered peptide ligands (APLs). As described in the implementation, we started with the crystal structure of a native gp34 LCMV epitope complexed within the mouse MHC class I molecule H-2K^b^. We introduced nitration into the p3TYR residue and refined the structure locally to resolve resulting clashes. The results were aligned and compared to the experimentally determined crystal structure. **A)** Orientation of the native gp34 in the resolved crystal structure [PDB: 3ROO]. The view is focused on the peptide-binding cleft. **B)** The refined model of a nitrated gp34 APL derived from the same crystal structure. Alternative backbone-dependent rotamers for GLU152 (steric displacement) and TYR116 (hydrogen bonding) have been chosen to accommodate this APL. **C)** The aligned gp34 APL and MHC pocket from the experimentally resolved crystal structure [PDB: 3ROL]. The essential adaptations are surprisingly similar. We therefore conclude that modeling APLs can be a valid predictive approach. However, the ligation of this APL induces more pronounced and global conformational changes that cannot be accounted for by local refinement. **D)** Relative positioning of the aligned peptides. Black and blue: Two nitrated gp34 epitopes according to modeling which correspond to 180° rotamers of each other. The first variant (model 1, black) clashed significantly with the helix of the MHC and TYR159, a feature that may contribute to the reduced affinity of the nitrated epitope (complete model not shown). The alternatively positioned model 2 was used for the modeling above and fits significantly better, especially after local refinement. Note how the orientation of this APL is essentially identical to that of the experimentally resolved variant (green).

As described in Figure 5D, we concluded that the orientation of the ortho-position, i.e. the rotamer of Nitro-tyrosine, is a factor that affects the affinity to the MHC. Steric clashes dictate that the nitro group only fits into the groove when facing away from the MHC helix into the groove. This problem does not occur for the Tyrosine itself. Furthermore, this positioning requires structural adaptation of GLU152. Consequently, the hydrogen bonding is altered for the nitrated gp34. The steric displacement of GLU152 breaks a hydrogen bond between this residue and the Nitrotyrosine hydroxyl group (Figure 5B). This loss is partially compensated for by TYR116 and GLN114 forming hydrogen bonds with the nitro group inside the groove. Taken together, this provides an explanation for the reduced binding capacity observed for the nitrated peptide. A final comparison of the refined model to the existing crystal structure with nitrated APL exhibited a striking similarity (Figure 5B vs. 5C).

Furthermore, in Additional file 3 we made use of the same model but introduced alternative PTMs, namely N-terminal acetylation (p1ACE-ALA) and/or methionine oxidation (p8SME). Based on the putative steric clashing with either the MHC rim (p1ACE-ALA) or the anchoring pocket (p8SME), we predict that these modifications will negate binding of the APL to H-2K^b^. The acetylated peptide is too long to fit the confinements of the MHC class I groove, and the anchoring pocket cannot properly accommodate the oxidized methionine. In summary, modeling of MHC-associated APLs is possible and yields predictive results within a reasonable frame of confidence. Larger global conformational changes, however, cannot be accounted for using this approach.

We conclude that modeling of APLs can be a useful approach prior to experimental investments. Furthermore, it can also be applicable to predict whether a PTM is likely to be in contact with the MHC and/or a corresponding T-cell receptor.

## Conclusions

### Perspective and future development

To summarize, we have presented several examples of how modeling using PyMOL and PyTMs can be applied to straightforwardly address various research questions related to PTMs. In either predictive or explanatory settings, modeling constitutes a rapid approach to investigate structural implications. The advantages of using a plugin to introduce PTMs include standardized generation of PTMs, rapid time-to-result and facilitated user control. In addition, modeling is an option to maximize cost-efficiency.

We identified two current limitations of the plugin, namely the incompatibility of non-standard residues with tested charge force field applications and a limited ability to assess changes in global conformation. Many PTMs are associated with drastic alterations in the charge pattern, rather than steric blocking, citrullination being a prime example. In concordance with this, conformational changes are probably a major mechanism through which certain PTMs mediate their effects. As both aspects are limited in PyTMS, this may restrict the interpretability of resulting models, unless well supported by experimental observations. Resolving these shortcomings presents a future challenge. Bearing this in mind, we support the reasonable application of *in silico* modeling alongside conventional structure determination.

In conclusion, PyTMs is a free, user-friendly and very convenient plugin for PyMOL and can be applied to straightforwardly address research questions connected to PTMs. We hope to address the discussed challenges over time and further extend the applicability with additional PTMs.

## Availability and requirements

**Project name(s):** PyTMs, PyMOL script repository.

**Project homepage:**

http://www.pymolwiki.org/index.php/Pytms.

https://github.com/Pymol-Scripts/Pymol-script-repo.

**Operating systems:** Platform independent.

**Programming language:** Python (https://www.python.org/).

**Other requirements:** PyMOL (http://www.pymol.org/).

**License:** GNU General Public License, version 2 (GPL-2.0).

## Additional files

Additional file 1:
**PyTMs.** The first release version of PyTMs (for deposit purposes); This file should optimally renamed to pytms.py and placed inside the ‘plugins’ folder of the ‘PyMOL-script-repo’ when installed. Note that PyTMs will be updated/maintained on the hompage site: http://www.pymolwiki.org/index.php/Pytms. Here you should find the latest version. Additional file 2:
**PyTMs supplementary images.** Supporting images, PyTMs menu screenshot and PTM overviews. Additional file 3:
**PyTMs supplementary online information.** Supporting information on the implementation of MDA/MAA adducts, supporting figures. Additional file 4:
**This is an example of the residue-based optimization in PyTMs.** The optimization resolves sterical clashes (represented by red discs) by positioning the adducted residue in a more favorable position, based on minimal local strain. This examples shows a test protein (PDB:1hpv) with malondialdehyde-acetaldehyde (MAA) adducts (red). The animated .gif file can be viewed in a web-browser. Additional file 5:
**PyTMs - graphical abstract.png.** Graphical abstract. In this example we modified a generic peptide (MKCRYKWKSTP) to display a selection of different modifications, as indicated. Colors: original carbons = white, hydrogens = light grey, nitrogens = blue, oxygens = red, sulfur= yellow, phosphorus = orange, adducted carbons = dark grey; MDA/MAA = malondialdehyde/ malondialdehyde-acetaldehyde.

## Abbreviations

API: Application programming interface
APL: Altered Peptide Ligand
MAA: Malondialdehyde Acetaldehyde
MDA: Malondialdehyde
MHC: Major Histocompatibility Complex
PTMs: Post-translational modifications
PyTMs: Name of the PyMOL plugin; an analogy to PyMOL and PTMs
TCR: T cell receptor
