# Supplementary material for: PyTMs: a useful PyMOL plugin for modeling common post-translational modifications
Source: BMC Bioinformatics. 2014 Nov 28;15(1):370. doi: 10.1186/s12859-014-0370-6 (PMC4256751; doi:10.1186/s12859-014-0370-6)

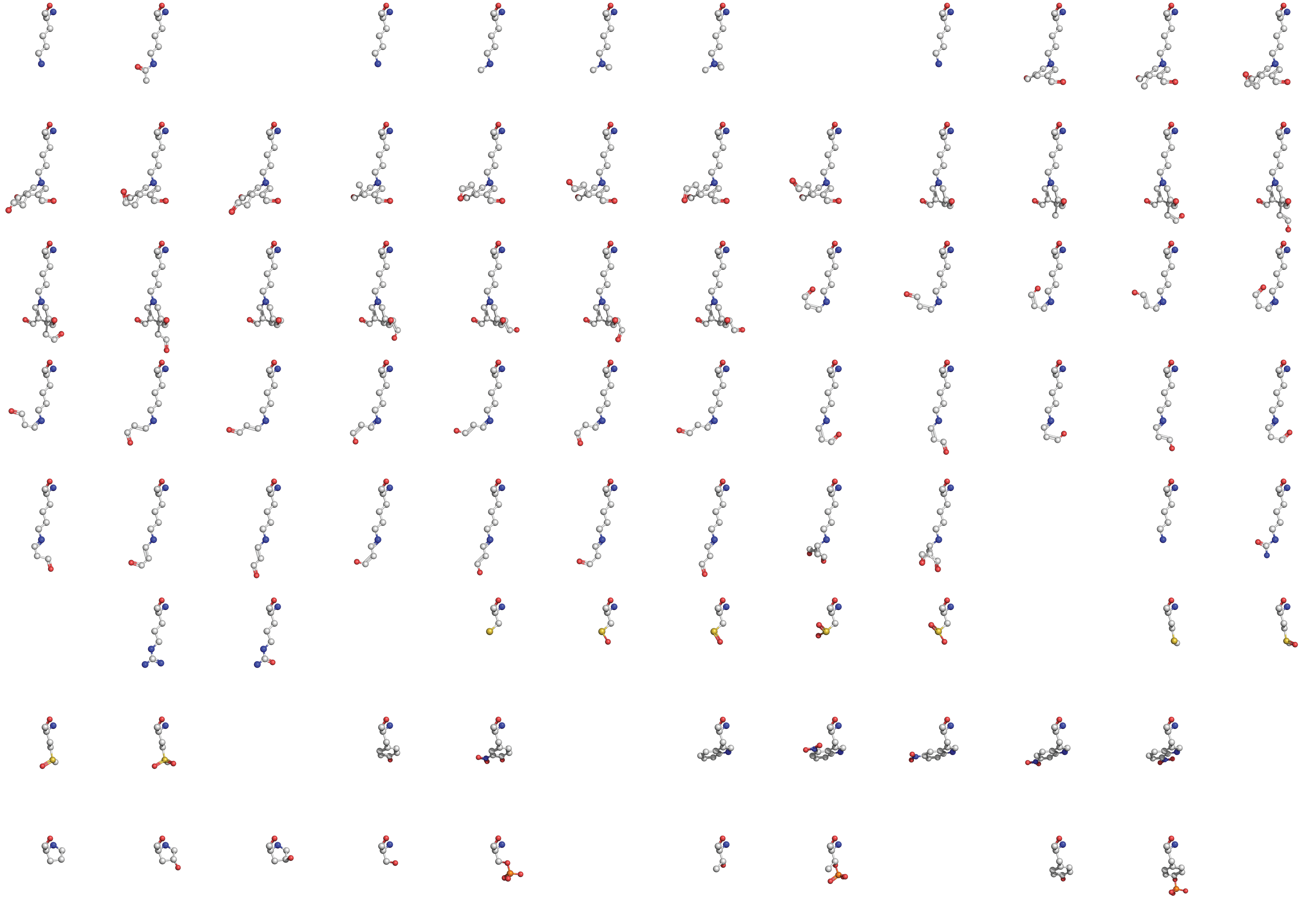

PyTMs: currently covered PTMs (colored by atom)

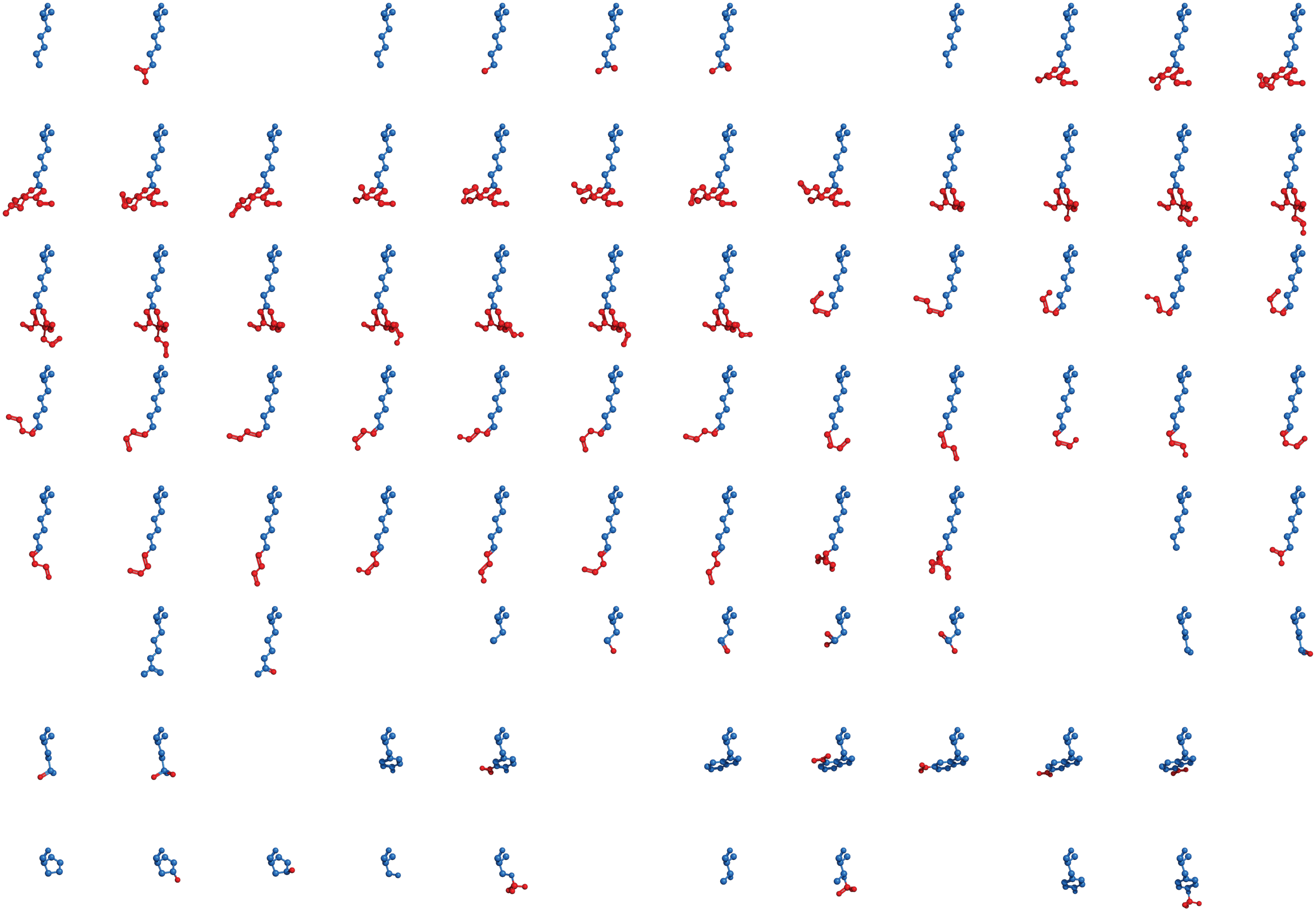

PyTMs: currently covered PTMs (colored by base/PTM)

PyTMs menu screenshot (not in paper)

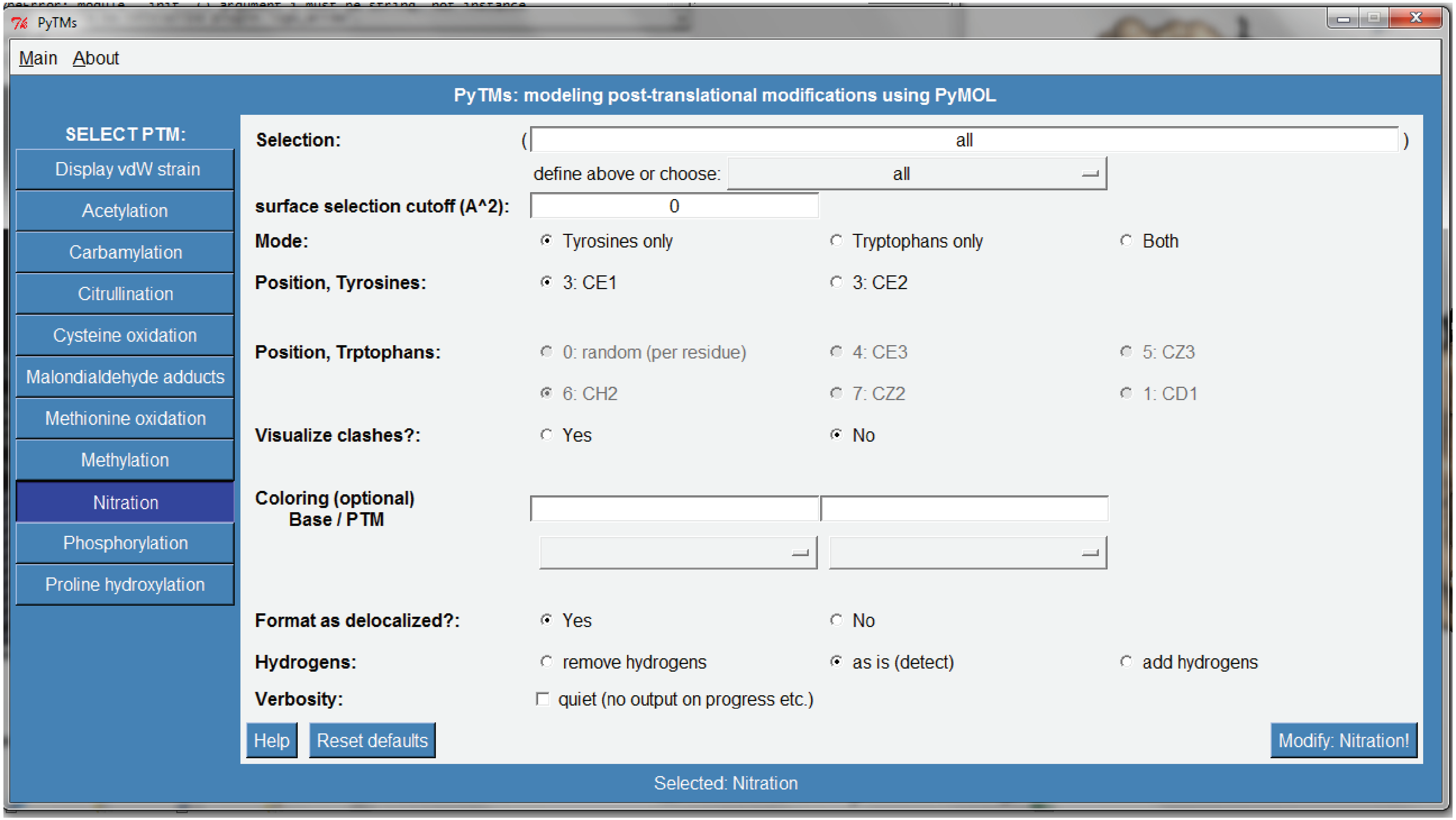

Supplement: Additional file 2: — PyTMs supplementary images. Supporting images, PyTMs menu screenshot and PTM overviews. [file 12859_2014_370_MOESM2_ESM.pdf]
