# Supplementary material for: PyTMs: a useful PyMOL plugin for modeling common post-translational modifications
Source: BMC Bioinformatics. 2014 Nov 28;15(1):370. doi: 10.1186/s12859-014-0370-6 (PMC4256751; doi:10.1186/s12859-014-0370-6)
Supplement: Additional file 3: — PyTMs supplementary online information. Supporting information on the implementation of MDA/MAA adducts, supporting figures. [file 12859_2014_370_MOESM3_ESM.pdf]

SUPPLEMENTARY INFORMATION AND FIGURE LEGENDS

Supplementary Information: MDA adduct implementation

In the conception of PyTMs, the absence of a resolved structure containing MDA-modified amino acids constituted a limitation. From the literature it became apparent that MDA does not form a distinct adduct but rather a spectrum composed of several categories and intermediate states. The categories include namely simple adducts [35, 36][1], dihydropyridine (DHP) type [31-33], the FAAB fragment [31] and crosslinks deriving from the previous variants [33, 34, 36]. Crosslinks have not been implemented in PyTMs. However, we implemented the MDA simple adducts, DHP type and FAAB fragment as separate hierarchical classes that further disseminate according to conformation and type (supplementary figure 1). In total it is possible to model 48 different MDA-related adducts which encompass both unique adducts and rotamers thereof (supplementary figure 1). Depending on the applied criteria, within the major groups individual variants may be partially redundant due to mesomeric/tautomeric forms, pseudo-isomers, rotamers or charge effects [48]. Others are plainly sterically unfavorable. Note that some MDA/MAA-adducts were distinguished for the sake of theoretic modeling and should be regarded as 'snapshots' rather than distinct variants [48]. The factual spectrum of MDA adducts is assumed to be more limited than the range that can be modeled. We analyzed the strain for the covered subtypes and concluded that *trans* isomers are clearly favorable, with little difference between the tautomeric variants (supplementary figure 1).

Whether the conformational variability of MDA adducts contributes to the proposed antigenicity reported for MDA-modified proteins is presently unclear. However, the single adducts, especially imine-type ones, are expected to be easily hydrolyzed at slightly basic pH and the biological half-life is therefore questionable. Accumulating evidence implies that the biologically most relevant MDA adducts are the Malondialdehyde-acetaldehyde (MAA) adducts, which belong to the DHP-type category. Furthermore, the strain analysis reveals that the trans-beta MAA adduct is among the most favorable. Moreover, a partial orbital hyper-conjugation explains the increased absorption at 280 nm and yellowish coloration (395 nm), as well as the loss in positive charge, despite being a tertiary amine. Experimentally, MDA-modified proteins have a lower pI value and increased negative charge ([36] and data not included). For the above-mentioned reasons, the MAA adduct is the default setting.

In the DHP-type category we included reaction products with formaldehyde, acetaldehyde or MDA itself as 'ring-closing' aldehyde [10, 33]. *In vitro*, however, the range of DHP-type derivates has been demonstrated to be somewhat larger, depending e.g. on the aldehydes present in cigarette smoke used during modification [10, 33]. As templates for the ring orientation we checked structure reports [49] and structures containing DHP-related compounds (e.g. [PDB: 2AMV]). From this we judged that the MAA adduct always adopts a boat configuration that could theoretically face towards the residue's ε-carbon (*cis*) or away (*trans*). Furthermore, the ring itself may be somewhat flexible, which is affected by the type of derivatization. We thus chose an average orientation in terms of torsions and angles, but included advanced settings that enable adjustment of the ring if deemed necessary. The derivates differ at the carbon 4 position, depending on the aldehyde present in the reaction. Starting with two-carbon aldehydes (and the resulting methyl group), configurational isomers in which the extension faces down

[1] The references correspond to the citations of the main article

from the boat (*alpha*) or up (*beta*) are conceivable. Though the modeling covers both, we analyzed the strain and conclude that the *beta* variant has a favorable energy as it does not clash with the adjacent carbaldehyde groups.

As MDA can not only modify epsilon amines of Lysine but any free amine, we added the option of modifying any N-terminus, with the exception of Proline. Which adduct variant is actually formed may depend on the experimental modification protocol used, and will likely yield a mixture.

Supplementary Figure 1: List of implemented MDA adducts

This supplementary figure gives an overview over the implemented MDA adducts.

Top: Table of MDA adduct as implemented in PyTMs

Adducts have been divided into three groups, namely: DHP-type (group=2), simple MDA adducts (group=3) or the FAAB-fragment (group=4). The second level is based on the adduct conformation (conformer), which determines the adduct orientation depending on the group (*cis/trans* or *alpha/beta*). Lastly, the type/tautomer ID will determine further configuration and tautomeric alternatives: in the DHP-type group this toggles the 4-carbon derivate, depending on the 'ring-closing' aldehyde (2: FA, 3:AA, 4-7:MDA). The 'type' variable toggles tautomeric forms in the simple MDA adduct group and format options for the FAAB adducts.

Rightmost column: The respective calculated vdW strains have been colored using a white-to-red spectrum by minimum-to-maximum values. Note how the *cis*-variants are associated with unfavorable strain. The default is a biologically relevant MAA adduct with relatively favorable strain (yellow highlight).

MDA: Malondialdehyde, AA: Acetaldehyde, FA: Formaldehyde; DHP: Dihydropyridine; MAA: Malondialdehyde-Acetaldehyde Adduct

Bottom:

B) A representation of selected DHP-type adducts derived from MDA exemplifying alternative configurations. The corresponding 3-digit number indicates group, conformer and type settings.

C) Overview of simple MDA tautomeric/rotameric variants. Note that most of these will be redundant for different reasons and should be regarded as 'snapshots' or intermediates. The settings are to be understood as a means of yielding a specific type and rotamer, rather than a unique adduct. Settings for conformer and type as indicated.

1. The structures are tentatively drawn with a charge and an ambiguous R2. This charge is the basis of R1-N isomers. Note that R2 may reflect another hydrogen or electron pair instead. Based on experimental results, however, we do not expect MDA-adducted Lysines to carry a positive charge.
2. The absence of double bonds, either between the nitrogen and the first MDA carbon (enamine/N-propenal variants) or between the adduct carbons (others) defines the resulting variants as rotamers. These rotamers were distinguished for the sake of modeling and are technically identical.
3. The tautomeric variants are expected to be in constant equilibrium.
4. Imine-type adducts can be hydrolyzed at basic pH and may not persist.
5. *cis*-isomers were calculated to be sterically unfavorable

Taken together, we implemented several MDA categories and variants that cover not only unique isomers but also rotamers thereof. The factual variety is expected to be more narrowly defined for the above-mentioned reasons. Whether this variability (in terms of category or conformation) is a factor contributing to the proposed antigenicity of MDA adducts remains to be explored.

G_ID	Group	Modification/ adduct	Abbreviation(s)	C_ID	Conformational isomer	T_ID	Type and/or Tautomer (position of oxygen, format)	Expected Mass increase [DA]	Optimized vdW strain (1 level)
NA	NA	Unmodified lysine residue	LYS	NA	NA	NA	NA	NA	93.09
0	Random (once)	Random (once)	Random (once)	0	Random (once)	0	Random (once)	Random (once)	NA
1	Random (by residue)	Random (by residue)	Random (by residue)	1	Random (by residue)	1	Random (by residue)	Random (by residue)	NA
2	DHP (MDA/FA)	2,6-dihyrdo-3,5-dicarbaldehyde-pyridine	DHDC	2 or 3	cis-boat	2	(Hydro) MFA	120.01	112.84
2	DHP (MDA/FA)	2,6-dihyrdo-3,5-dicarbaldehyde-pyridine	DHDC	4 or 5	trans-boat	2	(Hydro) MFA	120.01	102.57
2	DHP (MDA/AA)	4-methyl-2,6-dihyrdo-3,5-dicarbaldehyde-pyridine	MAA/MDHDC	2	cis-boat-alpha	3	Methyl (MAA)	134.13	308.39
2	DHP (MDA/MDA)	4-ethenol-2,6-dihyrdo-3,5-dicarbaldehyde-pyridine	EDHDC	2	cis-boat-alpha	4	Ethenol, O in cis	174.15	214.18
2	DHP (MDA/MDA)	4-ethenol-2,6-dihyrdo-3,5-dicarbaldehyde-pyridine	EDHDC	2	cis-boat-alpha	5	Ethenol, O in trans	174.15	193.05
2	DHP (MDA/MDA)	4-ethanal-2,6-dihyrdo-3,5-dicarbaldehyde-pyridine	EDHDC	2	cis-boat-alpha	6	Ethanal, O in cis	174.15	318.42
2	DHP (MDA/MDA)	4-ethanal-2,6-dihyrdo-3,5-dicarbaldehyde-pyridine	EDHDC	2	cis-boat-alpha	7	Ethanal, O in trans	174.15	302.12
2	DHP (MDA/AA)	4-methyl-2,6-dihyrdo-3,5-dicarbaldehyde-pyridine	MAA/MDHDC	3	cis-boat-beta	3	Methyl (MAA)	134.13	155.17
2	DHP (MDA/MDA)	4-ethenol-2,6-dihyrdo-3,5-dicarbaldehyde-pyridine	EDHDC	3	cis-boat-beta	4	Ethenol, O in cis	174.15	144.88
2	DHP (MDA/MDA)	4-ethenol-2,6-dihyrdo-3,5-dicarbaldehyde-pyridine	EDHDC	3	cis-boat-beta	5	Ethenol, O in trans	174.15	116.22
2	DHP (MDA/MDA)	4-ethanal-2,6-dihyrdo-3,5-dicarbaldehyde-pyridine	EDHDC	3	cis-boat-beta	6	Ethanal, O in cis	174.15	153.66
2	DHP (MDA/MDA)	4-ethanal-2,6-dihyrdo-3,5-dicarbaldehyde-pyridine	EDHDC	3	cis-boat-beta	7	Ethanal, O in trans	174.15	121.41
2	DHP (MDA/AA)	4-methyl-2,6-dihyrdo-3,5-dicarbaldehyde-pyridine	MAA/MDHDC	4	trans-boat-alpha	3	Methyl (MAA)	134.13	236.44
2	DHP (MDA/MDA)	4-ethenol-2,6-dihyrdo-3,5-dicarbaldehyde-pyridine	EDHDC	4	trans-boat-alpha	4	Ethenol, O in cis	174.15	207.28
2	DHP (MDA/MDA)	4-ethenol-2,6-dihyrdo-3,5-dicarbaldehyde-pyridine	EDHDC	4	trans-boat-alpha	5	Ethenol, O in trans	174.15	181.91
2	DHP (MDA/MDA)	4-ethanal-2,6-dihyrdo-3,5-dicarbaldehyde-pyridine	EDHDC	4	trans-boat-alpha	6	Ethanal, O in cis	174.15	325.21
2	DHP (MDA/MDA)	4-ethanal-2,6-dihyrdo-3,5-dicarbaldehyde-pyridine	EDHDC	4	trans-boat-alpha	7	Ethanal, O in trans	174.15	307.02
**2**	**DHP (MDA/AA)**	**4-methyl-2,6-dihyrdo-3,5-dicarbaldehyde-pyridine**	**MAA/MDHDC**	**5**	**trans-boat-beta**	**3**	**Methyl (MAA)**	**134.13**	**102.68**
2	DHP (MDA/MDA)	4-ethenol-2,6-dihyrdo-3,5-dicarbaldehyde-pyridine	EDHDC	5	trans-boat-beta	4	Ethenol, O in cis	174.15	142.70
2	DHP (MDA/MDA)	4-ethenol-2,6-dihyrdo-3,5-dicarbaldehyde-pyridine	EDHDC	5	trans-boat-beta	5	Ethenol, O in trans	174.15	111.12
2	DHP (MDA/MDA)	4-ethanal-2,6-dihyrdo-3,5-dicarbaldehyde-pyridine	EDHDC	5	trans-boat-beta	6	Ethanal, O in cis	174.15	142.07
2	DHP (MDA/MDA)	4-ethanal-2,6-dihyrdo-3,5-dicarbaldehyde-pyridine	EDHDC	5	trans-boat-beta	7	Ethanal, O in trans	174.15	111.09
3	MDA	N-propenal	MDA	2	R1-N cis/ C1-C2 cis	2	enamine, O in cis	54.05	623.37
3	MDA	N-propenal	MDA	2	R1-N cis/ C1-C2 cis	3	enamine, O in trans	54.05	473.48
3	MDA	N-iminopropenol	MDA	2	R1-N cis/ C1-C2 cis	4	imine-enol, O in cis	54.05	595.06
3	MDA	N-iminopropenol	MDA	2	R1-N cis/ C1-C2 cis	5	imine-enol, O in trans	54.05	486.40
3	MDA	N-iminopropanal	MDA	2	R1-N cis/ C1-C2 cis	6	imine-al, O in cis	54.05	628.02
3	MDA	N-iminopropanal	MDA	2	R1-N cis/ C1-C2 cis	7	imine-al, O in trans	54.05	523.41
3	MDA	N-propenal	MDA	3	R1-N cis/ C1-C2 trans	2	enamine, O in cis	54.05	217.84
3	MDA	N-propenal	MDA	3	R1-N cis/ C1-C2 trans	3	enamine, O in trans	54.05	227.30
3	MDA	N-iminopropenol	MDA	3	R1-N cis/ C1-C2 trans	4	imine-enol, O in cis	54.05	198.27
3	MDA	N-iminopropenol	MDA	3	R1-N cis/ C1-C2 trans	5	imine-enol, O in trans	54.05	205.25
3	MDA	N-iminopropanal	MDA	3	R1-N cis/ C1-C2 trans	6	imine-al, O in cis	54.05	165.84
3	MDA	N-iminopropanal	MDA	3	R1-N cis/ C1-C2 trans	7	imine-al, O in trans	54.05	171.84
3	MDA	N-propenal	MDA	4	R1-N trans/ C1-C2 cis	2	enamine, O in cis	54.05	170.15
3	MDA	N-propenal	MDA	4	R1-N trans/ C1-C2 cis	3	enamine, O in trans	54.05	147.39
3	MDA	N-iminopropenol	MDA	4	R1-N trans/ C1-C2 cis	4	imine-enol, O in cis	54.05	407.19
3	MDA	N-iminopropenol	MDA	4	R1-N trans/ C1-C2 cis	5	imine-enol, O in trans	54.05	222.55
3	MDA	N-iminopropanal	MDA	4	R1-N trans/ C1-C2 cis	6	imine-al, O in cis	54.05	173.02
3	MDA	N-iminopropanal	MDA	4	R1-N trans/ C1-C2 cis	7	imine-al, O in trans	54.05	234.11
3	MDA	N-propenal	MDA	5	R1-N trans/ C1-C2 trans	2	enamine, O in cis	54.05	132.14
3	MDA	N-propenal	MDA	5	R1-N trans/ C1-C2 trans	3	enamine, O in trans	54.05	131.99
3	MDA	N-iminopropenol	MDA	5	R1-N trans/ C1-C2 trans	4	imine-enol, O in cis	54.05	111.36
3	MDA	N-iminopropenol	MDA	5	R1-N trans/ C1-C2 trans	5	imine-enol, O in trans	54.05	106.54
3	MDA	N-iminopropanal	MDA	5	R1-N trans/ C1-C2 trans	6	imine-al, O in cis	54.05	106.40
3	MDA	N-iminopropanal	MDA	5	R1-N trans/ C1-C2 trans	7	imine-al, O in trans	54.05	106.54
4	FAAB	2-formyl-3-(alkylamino)butanal	FAAB	2,4	R configuration	2,5	delocalized bonds, closed	96.08	165.10
4	*FAAB*	*2-formyl-3-(alkylamino)butanal*	*FAAB*	*2,4*	*R configuration*	*3,6*	*delocalized bonds, open*	*96.08*	*165.10*
4	*FAAB*	*2-formyl-3-(alkylamino)butanal*	*FAAB*	*2,4*	*R configuration*	*4,7*	*regular bonds (open)*	*96.08*	*165.10*
4	FAAB	2-formyl-3-(alkylamino)butanal	FAAB	3,5	S configuration	2,5	delocalized bonds, closed	96.08	167.06
4	*FAAB*	*2-formyl-3-(alkylamino)butanal*	*FAAB*	*3,5*	*S configuration*	*3,6*	*delocalized bonds, open*	*96.08*	*167.06*
4	*FAAB*	*2-formyl-3-(alkylamino)butanal*	*FAAB*	*3,5*	*S configuration*	*4,7*	*regular bonds (open)*	*96.08*	*167.06*

B)

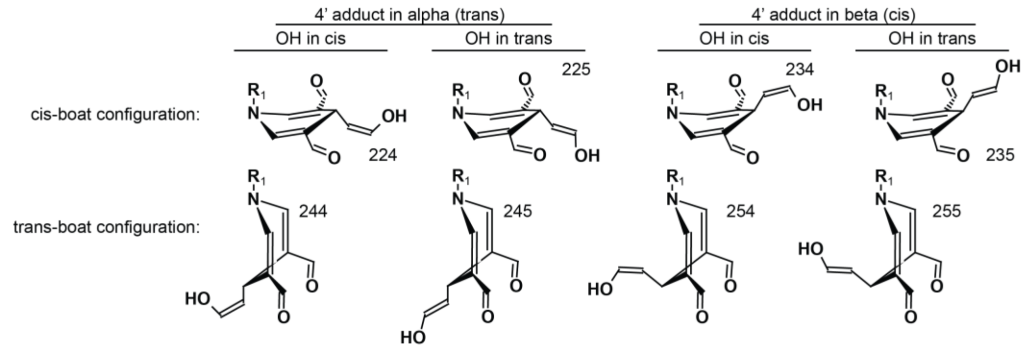

C)

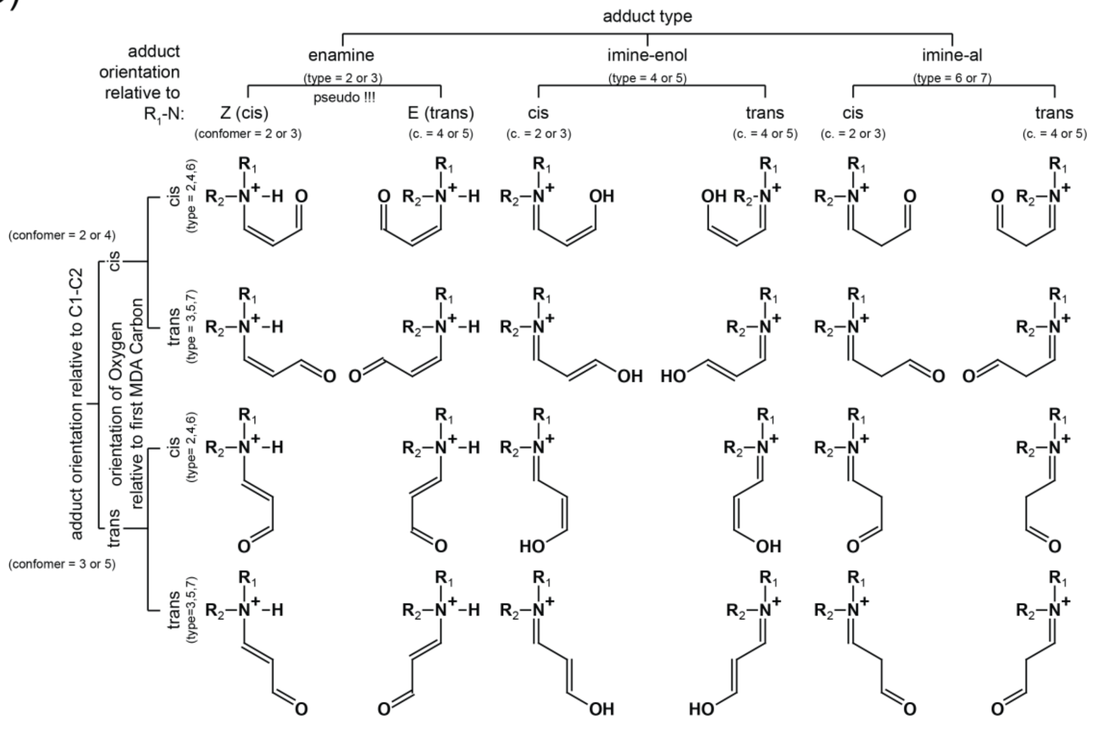

Supplementary Figure 2: MHC–related applications

This supplementary figure relates to Figure 5 of the main article. Here, instead of nitration, we modeled N-terminal acetylation of p1ALA and oxidation of p8MET. The p1ALA is positioned at the rim of the MHC pocket and p8MET is an anchoring residue. Both modifications mediate significant steric displacement. We thus predict that either modification will result in the modified gp34 epitope being a non-binding variant.

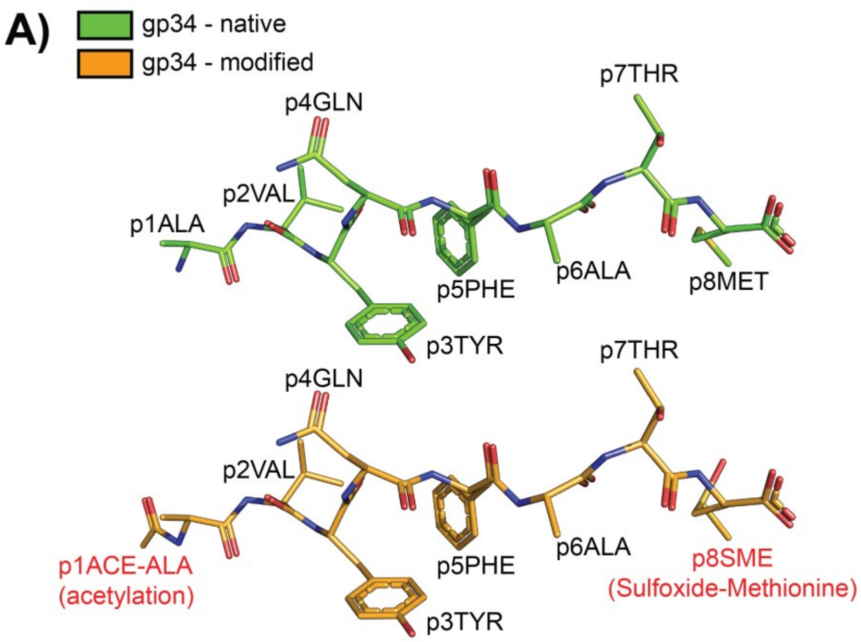

A) Labeled display of the native (green) peptide and a double-modified variant (orange) in the same orientation.

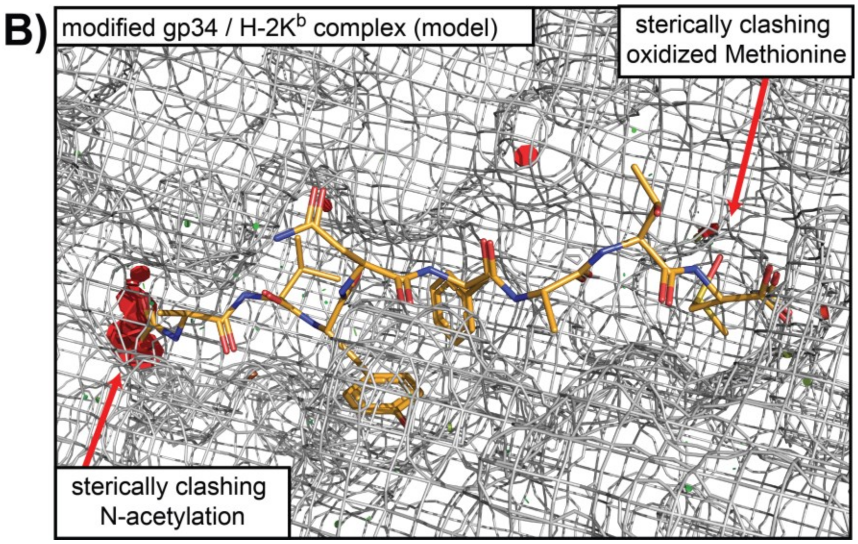

B) The modified variant modeled inside the native H-2K^b. The surface extent is outlined using a mesh representation. Steric clashes are indicated by the red discs. We conclude that this variant (or either single variant) will not be able to stably bind.
N-terminal acetylation of p1-ALA elongates the peptide ligand and clashes with the MHC rim (TRP167 and TYR59, TYR171). We conclude that the acetyl group cannot be rotated to a fitting position and that the acetylated peptide is too long for the confinements of the H-2K^b (MHC class I) groove.
The pocket of p8MET is mainly lined by hydrophobic residues: ILE95, LEU81, TYR116, TYR123 and a negatively charged ASP77 closest to the hypothetical oxidation (not depicted). All tested rotamers for ASP77 clashed with either the Methionine sulfoxide (SME) or neighboring MHC residues. We therefore conclude that a more polar and larger Methionine sulfoxide may not fit the p8 pocket constrains.
