# Supplementary figures and images for: PyTMs: a useful PyMOL plugin for modeling common post-translational modifications
Source: BMC Bioinformatics. 2014 Nov 28;15(1):370. doi: 10.1186/s12859-014-0370-6 (PMC4256751; doi:10.1186/s12859-014-0370-6)

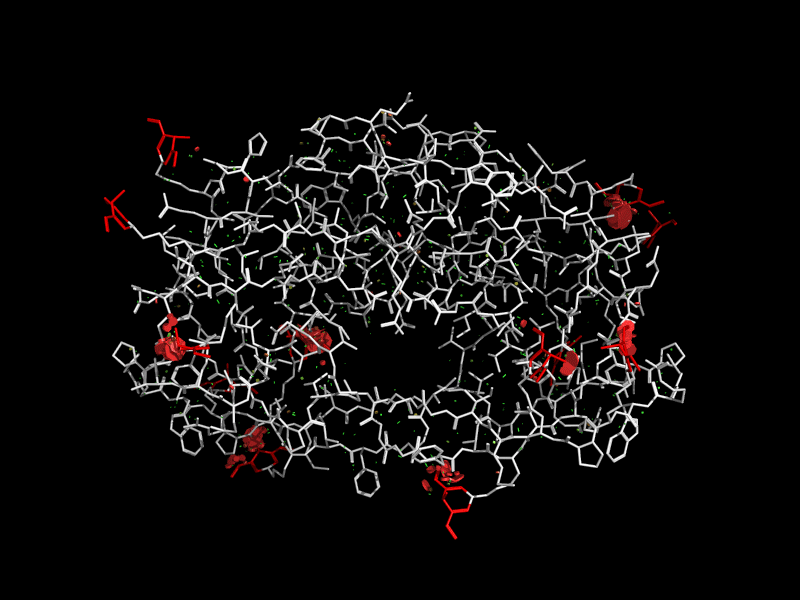

Supplement: Additional file 4: — This is an example of the residue-based optimization in PyTMs. The optimization resolves sterical clashes (represented by red discs) by positioning the adducted residue in a more favorable position, based on minimal local strain. This examples shows a test protein (PDB:1hpv) with malondialdehyde-acetaldehyde (MAA) adducts (red). The animated .gif file can be viewed in a web-browser. [file 12859_2014_370_MOESM4_ESM.gif]

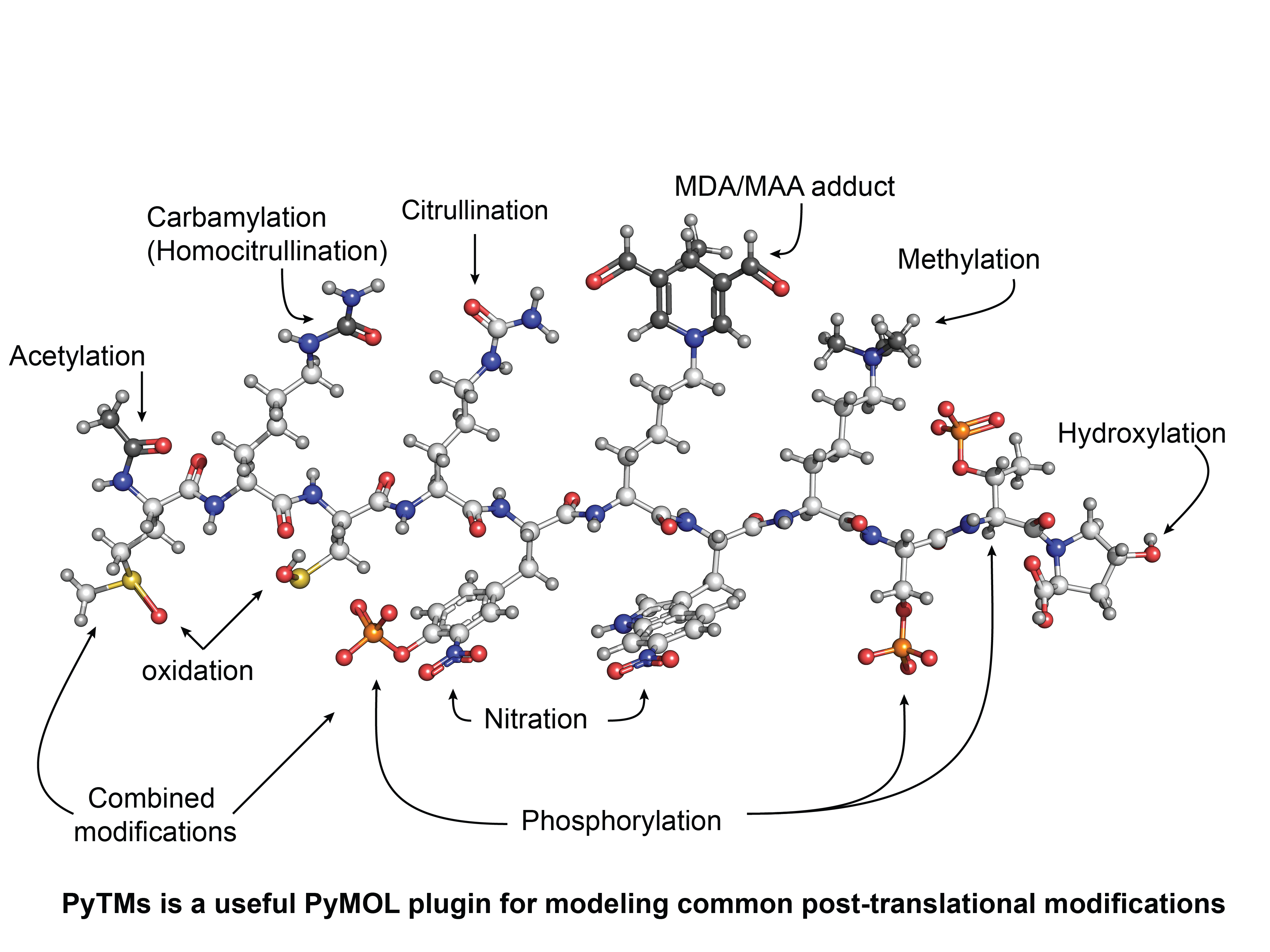

Supplement: Additional file 5: — PyTMs - graphical abstract.png. Graphical abstract. In this example we modified a generic peptide (MKCRYKWKSTP) to display a selection of different modifications, as indicated. Colors: original carbons = white, hydrogens = light grey, nitrogens = blue, oxygens = red, sulfur= yellow, phosphorus = orange, adducted carbons = dark grey; MDA/MAA = malondialdehyde/ malondialdehyde-acetaldehyde. [file 12859_2014_370_MOESM5_ESM.png]
